# Case report: Uniportal video-assisted thoracoscopic sleeve lobectomy in a 6-year-old patient with inflammatory myofibroblastic tumor (IMT)

**DOI:** 10.3389/fped.2023.1285181

**Published:** 2023-10-17

**Authors:** Zhen-Yang Geng, Zi-Hao Li, Shi-Hao Li, Bin Wu, Yin-Liang Sheng, Ping Yuan, Feng Li, Yu Qi

**Affiliations:** Department of Thoracic Surgery, First Affiliated Hospital of Zhengzhou University, Zhengzhou, China

**Keywords:** inflammatory myofibroblastic tumor, thoracic surgery, uniportal thoracoscopic, sleeved lobectomy, pediatric patients

## Abstract

Inflammatory myofibroblastic tumor (IMT) is a rare neoplasm that can occur in various organs, including the lung. Surgical resection is usually the preferred treatment for localized IMT.A 6-year-old female was admitted to our hospital with complaints of “coughing and vomiting for 6 days”. A chest CT scan revealed occlusion of the left main bronchus, segmental atelectasis of the left lower lung, and cystic low-density shadows along the bronchial pathway. Subsequent fiberoptic bronchoscopy confirmed the diagnosis of IMT through pathological biopsy. After excluding surgical contraindications, the patient underwent uniportal video-assisted thoracoscopic sleeve lobectomy for treatment. The patient had an uneventful postoperative course and was discharged four days after surgery. After one month, the patient received a follow-up examination and reported no significant discomfort. A chest CT scan revealed no postoperative complications.Our experience suggests that uniportal video-assisted thoracoscopic surgery may be a safe and effective approach for the treatment of pediatric patients with IMT requiring complex surgical procedures such as sleeve lobectomy and tracheoplasty.

## Introduction

Inflammatory myofibroblastic tumor (IMT) is a rare mesenchymal tumor that frequently occurs in children and young adults. Although it can arise in multiple organs, it is commonly found in the lungs (1). Primary lung tumors are relatively uncommon in pediatric patients, with pulmonary IMT accounting for approximately 50% of benign primary lung tumors in this population and representing the most common type of primary lung tumor in children (2). To mitigate the risk of local recurrence, it is essential to implement complete surgical resection during treatment (2). Patients who undergo such surgical intervention generally enjoy a satisfactory prognosis, with a 5-year survival rate of over 90% (3, 4). This article reports a case of a very young patient who underwent sleeve resection of the lung lobe via a single-hole thoracoscopy, representing a pioneering approach to the treatment of pediatric lung surgery.

## Case presentation

**Figure 1:**
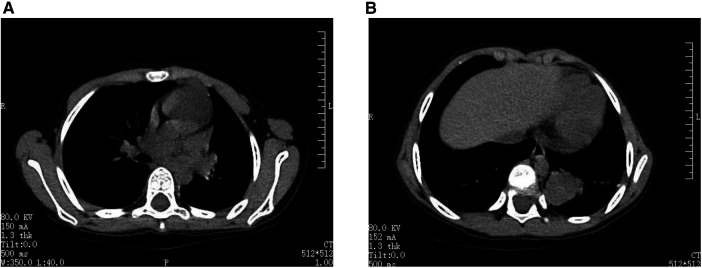
Chest CT of the patient. (**A**) Preoperative examination before bronchoscopy. CT scan revealed occlusion of the left main bronchus. (**B**) An irregular soft tissue mass was observed in the lower left lung.

A 6-year-old female presented with intermittent coughing episodes that occurred irregularly once every year, each episode lasting for about 2 days. The patient possessed a prior medical history involving asphyxia at the time of birth, accompanied by an indeterminate Apgar score, and subsequently received treatment for “aspiration pneumonia” at a nearby medical facility. The child was admitted to our hospital with complaints of “coughing and vomiting for 6 days”. Upon admission, a chest CT scan revealed occlusion of the left main bronchus, segmental atelectasis of the left lower lung, and cystic low-density shadows along the bronchial pathway (Figure 1).

**Figure 2:**
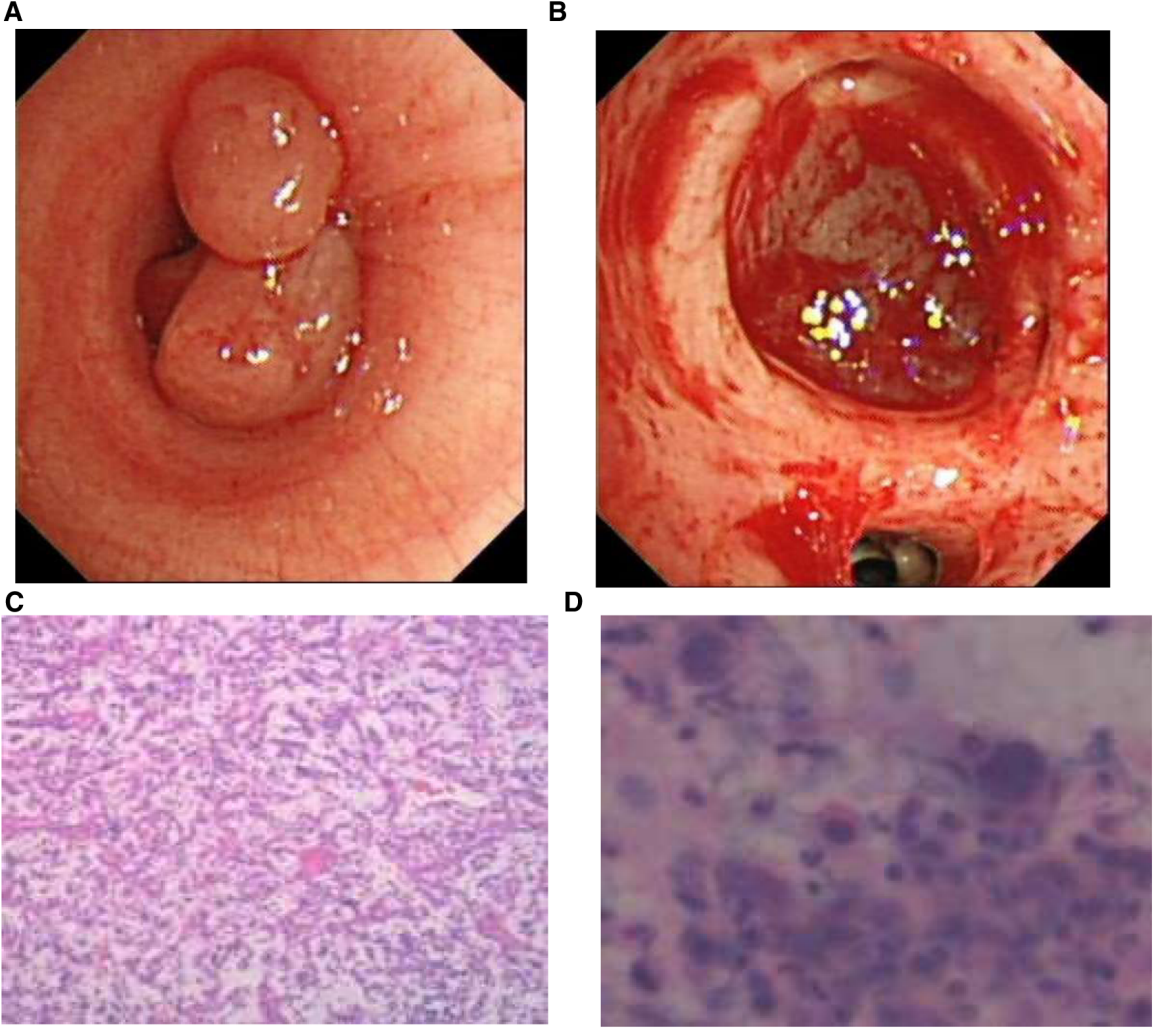
(**A**) Bronchoscopy revealed a polypoid neoplasm that nearly completely obstructed the lumen of the left main bronchus. (**B**) After resection of the intratracheal mass, the distal part of the basal segment of the left lower lung remained obstructed. (**C**) The pathological report indicated an inflammatory myofibroblastic tumor. (**D**) Nuclear pleomorphism was also observed in the lavage fluid.

Fiber bronchoscopy was performed, and it revealed the presence of polypoid neoplasms in the left main bronchus, which almost completely obstructed the lumen (Figure 2A). To provide both treatment and diagnosis, high-frequency electrocautery snare was applied to remove the masses in the left main bronchus and perform cryotherapy. Even after treatment, it could be seen that the distal left lower lobe basal segment remained obstructed (Figure 2B).

We sent the excised tissue for pathological examination, washed the left lower lobe of the lung with physiological saline, and then analyzed the extracted lavage solution.Histological examination of the excised specimen revealed scattered large cells with prominent nucleoli and occasional mitoses in the background of chronic active inflammation, suggestive of an inflammatory myofibroblastic tumor (Figures 2C,D).

Immunohistochemical tests displayed the following results: AE1/AE3 (-), EMA (-), CD68 (-), CD207 (-), CD15 (-), S-100 (-), SOX-10 (-), CD20 (+), CD3 (+), CD30 (-), MPO (-), ALK (5A4) (-), CD34 (-), CD31 (-), SMA (-), Desmin (-), INI-1 (+), SMARCA4 (+), SALL4 (-), and Ki-67 (+). To confirm the diagnosis, We sent the slices to the Shanghai Akman Medical Research Institute for consultation outside the hospital. Cultures from bronchoalveolar lavage were normal. During this period, the patient was treated symptomatically with anti-inflammatory drugs. The preliminary diagnosis was confirmed by results of the out-of-hospital consultation. To assess the patient's overall condition further, a PET-CT examination was performed, which revealed no other suspected hypermetabolic lesions. After excluding relevant surgical contraindications, surgical treatment was determined to be the best course of action.

The patient was placed in the right lateral position under general anesthesia, tracheal double-lumen intubation, and a central line was inserted in the internal jugular vein. A single 4-cm incision was made on the fifth intercostal space of the left midaxillary line, and an incision protector was placed to safeguard the incision site.

Thoracoscopy was employed to explore the chest cavity through the incision, revealing partial membranous adhesion in the left chest cavity. Notably, no clearly enlarged lymph nodes were detected near the bronchus or below the carina. Post-exploration, an electric hook was used to burn off the pleural adhesions.

**Figure 3:**
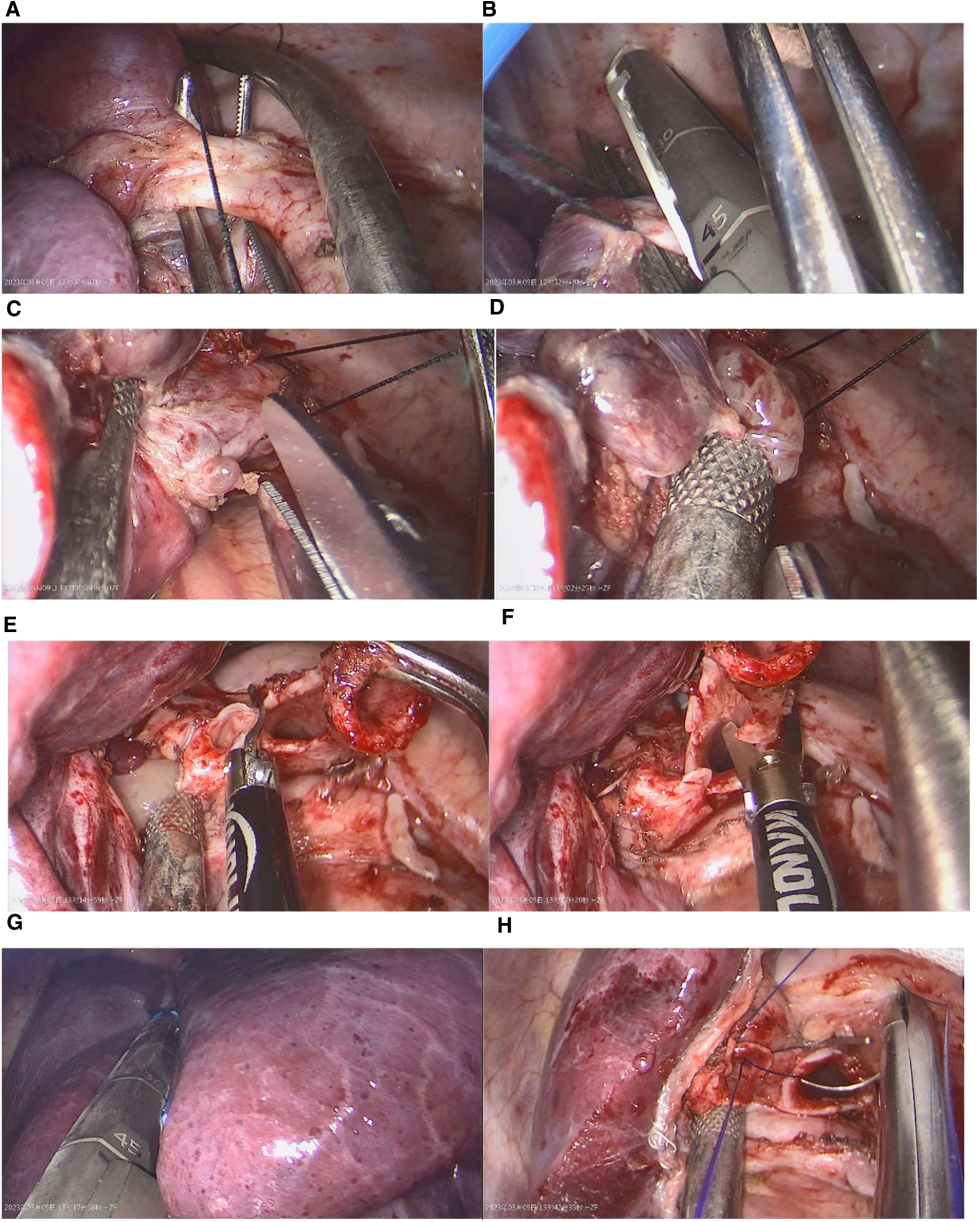
Key surgical steps: (**A**,**B**) After dissecting the pulmonary ligament, the left lower pulmonary vein was cut and sutured using a linear cutting device. (**C**,**D**) The proximal portion of the left lower bronchus was incised, and its contents were suctioned. (**E**,**F**) The proximal portion of the left upper bronchus was severed, and the left main bronchus was cut from its distal end. (**G**) The left lower lobe was excised using a linear cutting device. (**H**) Finally, the bronchial resection margin were trimmed, and the two ends were sutured continuously using absorbable sutures.

To facilitate the dissection of the left lower pulmonary vein, the lower lung ligament was disconnected, and a linear cutting and sewing machine was utilized for incision closure. In subsequent steps, we carefully freed the left lower lobe and main bronchus, followed by using a laparoscopic scissors to remove the distal of the left main bronchus and the proximal of the left upper lobe bronchus. Finally, the left lower lobe was excised. The remaining soft tissue-like content in the bronchus were exposed, and a suction device was utilized to extract them. After removal of the left lower lobe of the lung, the distal end of the left main bronchus and the proximal end of the left upper lobe bronchus were subsequently sutured together continuously with a 3-0 sliding thread. Additionally, thoracic lymph nodes were methodically cleared (Figure 3).

In the final stage of the operation, the chest cavity was irrigated with physiological saline, and the anastomosis was examined. The remaining lung lobe expanded properly (at a ventilation pressure of 25cmH_2_O), and no bronchial stump leakage was observed. Following hemostasis at each operating site, a closed drainage tube was positioned in the 5th intercostal space along the left midaxillary line, and the wound was closed.

The surgical operation lasted for 159 min, and the patient had minimal blood loss, which did not require a blood transfusion. Once the patient awoke in the recovery room, admission to the ICU was not necessary, and no complications were reported at weaning from sedation. and the patient was subsequently moved to the ward. Following the operation, histopathological analysis confirmed R0 at examination and no regional lymph node metastasis.

**Figure 4:**
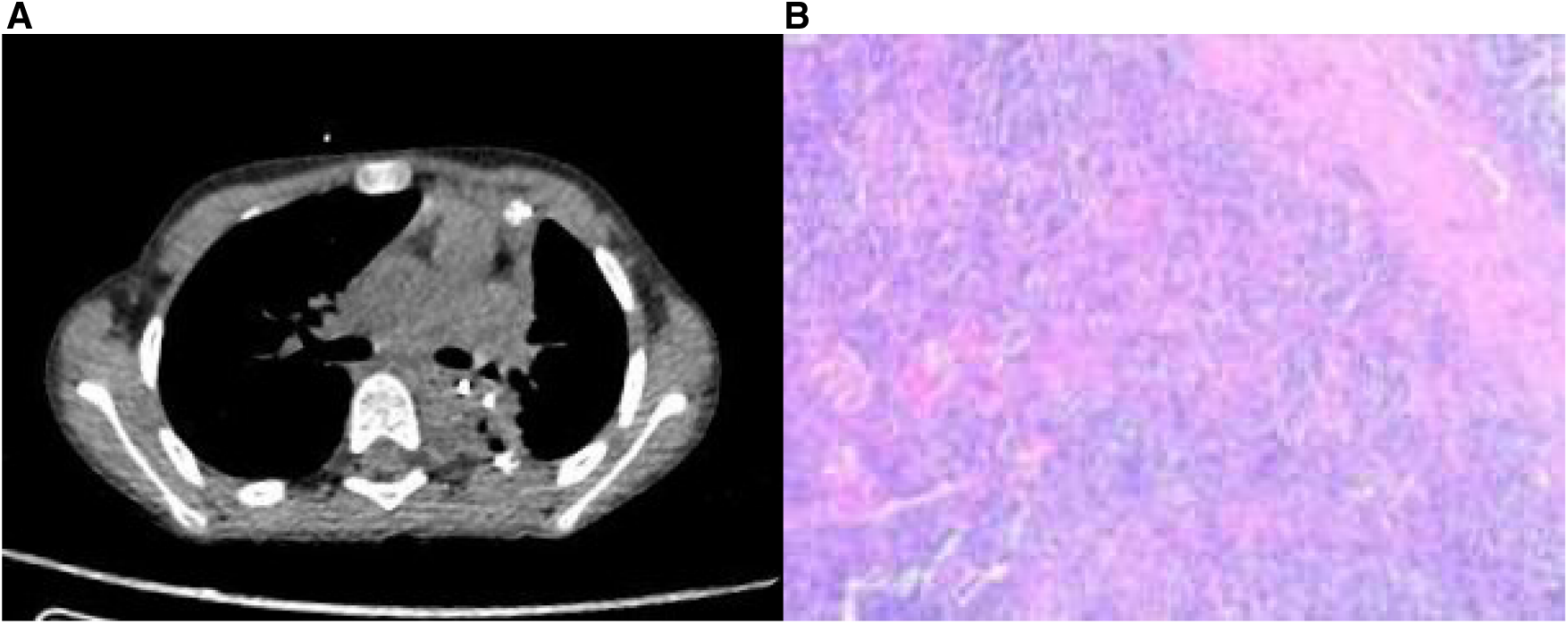
(**A**) Postoperative CT scan revealed a patent airway without any apparent surgical complications. (**B**) Histopathological examination of the postoperative specimen demonstrated increased thin-walled blood vessels and a few scattered large cells within an inflammatory cellular background.

The patient recovered well with no respiratory symptoms observed. A chest CT scan showed satisfactory expansion of the remaining lung lobes, mild local inflammation, and a small amount of pleural effusion. The drainage tube was removed on the fifth day post-operation, and the patient was discharged one day later (Figure 4). After one month, the patient received a follow-up examination and reported no significant discomfort. A chest CT scan revealed no postoperative complications.

## Discussion

In children, IMT in the lungs more commonly occurs in the peripheral area, displaying slow growth patterns and local invasiveness (2). The clinical manifestations of IMT often lack specificity, with solitary lung masses being the primary imaging characteristic (5). As a result, there can be significant challenges in accurately diagnosing IMT. Fine needle aspiration and fiber bronchoscopy exams are often unsuccessful due to limited sample availability (6, 7). In many cases, clinicians must employ surgical methods to remove tissue samples for pathological diagnoses. Immunohistochemical techniques are commonly used for diagnosing IMT (8). The presentation of atelectasis in this patient resulting from tumor obstruction of the main bronchus is relatively uncommon (9). Therefore, bronchoscopy can be employed to obtain more tissues for a biopsy to achieve an accurate diagnosis.

Because of the potential multifocal nature of IMT, a patient's entire condition must be assessed systematically prior to selecting a treatment plan. A PET-CT scan offers a comprehensive evaluation of metabolic activity among organs and tissues. The scan identifies isolated masses and can further help determine the presence or absence of distant metastatic lesions (10). For localized pulmonary IMT, complete surgical resection remains the primary treatment method to prevent recurrence (11).

The choice of surgical method for treating IMT depends on the specific range of lesions, with common procedures including lobectomy and pneumonectomy. For this pediatric patient, we opted for sleeve lobectomy to completely remove the lesion. Numerous published studies suggest that the majority of pediatric patients with pulmonary IMT who require surgical treatment undergo thoracotomy, rather than minimally invasive thoracoscopic procedures. Compared to thoracotomy, television-assisted thoracoscopic surgery offers multiple benefits, such as improved postoperative recovery and reduced harm to the patient (12, 13). Over the past 20 years, single hole thoracoscopy has rapidly developed and has gradually replaced traditional three-hole thoracoscopy as the mainstream technique due to its capacity to induce less trauma. However, it remains infrequently applied in pediatric care, likely due to the narrow thoracic cavity of pediatric patients and the absence of specialized pediatric endoscopic equipment (14). Hence, it is imperative for doctors with adequate and specialized training to perform these procedures.

There is currently no consensus about whether lymph node dissection is necessary for patients with pulmonary IMT. However, reports indicate that mediastinal lymph node involvement was present in some patients with PIMT undergoing intraoperative resection (15). Additionally, some researchers suggest lymph node dissection for confirmed IMT cases (16). In this specific case, the patient's IMT diagnosis had been established before undergoing surgery; accordingly, lymph node dissection was performed, and no abnormalities were detected.

For resectable lesions, surgical treatment enjoys a position of superiority (11, p. 3 of 8). Nonetheless, there is a possibility of local recurrence following complete resection, even after an extended period of time (6). Even when experiencing a local recurrence, adjuvant therapy is not the preferred treatment, and surgical intervention is recommended to prolong survival, provided the patient's general condition allows for it (15). Therefore, long-term monitoring and follow-up examinations are necessary for high-risk patients (17). Research also suggests that the outcomes for simple surgical treatment and surgical treatment combined with adjuvant therapies, such as radiotherapy and chemotherapy, are not significantly different (18). Adjuvant therapy is generally employed for IMT patients either with incomplete resection or other surgical contraindications (17).

To conclude, pulmonary IMT is a rare disease that is generally treated with surgery. The 6-year-old patient, who was 113 cm tall and weighed 19 kg, was the youngest case to have undergo single-hole thoracoscopic sleeve lung resection, and our experience has demonstrated the viability of this technique for young children with pulmonary IMT.

## Data availability statement

The original contributions presented in the study are included in the article/Supplementary Material, further inquiries can be directed to the corresponding author.

## Ethics statement

Written informed consent was obtained from the patients for the publication of any potentially identifiable images or data in this article.

## Author contributions

Z-YG: Writing—original draft, Writing – review & editing. Z-HL: Writing – review & editing. S-HL: Writing – review & editing. Y-LS: Writing – review & editing. PY: Writing – review & editing. FL: Writing – review & editing. YQ: Supervision, Writing – review & editing.

## Funding

The author(s) declare financial support was received for the research, authorship, and/or publication of this article.

This work was supported by the funds from the Cultivation Project of Henan Health Science and Technology Innovation Talents (Grant No. YXKC2022014, YQRC2023011) and the Henan Provincial Science and Technology Development Project (Grant No. 222102310239, LHGJ20210286).

## Conflict of interest

The authors declare that the research was conducted in the absence of any commercial or financial relationships that could be construed as a potential conflict of interest.

## Publisher's note

All claims expressed in this article are solely those of the authors and do not necessarily represent those of their affiliated organizations, or those of the publisher, the editors and the reviewers. Any product that may be evaluated in this article, or claim that may be made by its manufacturer, is not guaranteed or endorsed by the publisher.
